# Biomedical Application of Enzymatically Crosslinked Injectable Hydrogels

**DOI:** 10.3390/gels10100640

**Published:** 2024-10-07

**Authors:** Minho Nam, Jong Won Lee, Gi Doo Cha

**Affiliations:** Department of Systems Biotechnology, Chung-Ang University, Anseong 17546, Republic of Korea; skaalsgh2000@cau.ac.kr (M.N.); ljw0971@cau.ac.kr (J.W.L.)

**Keywords:** injectable hydrogel, enzymatic crosslinking, biomedical application

## Abstract

Hydrogels have garnered significant interest in the biomedical field owing to their tissue-like properties and capability to incorporate various fillers. Among these, injectable hydrogels have been highlighted for their unique advantages, especially their minimally invasive administration mode for implantable use. These injectable hydrogels can be utilized in their pristine forms or as composites by integrating them with therapeutic filler materials. Given their primary application in implantable platforms, enzymatically crosslinked injectable hydrogels have been actively explored due to their excellent biocompatibility and easily controllable mechanical properties for the desired use. This review introduces the crosslinking mechanisms of such hydrogels, focusing on those mediated by horseradish peroxidase (HRP), transglutaminase (TG), and tyrosinase. Furthermore, several parameters and their relationships with the intrinsic properties of hydrogels are investigated. Subsequently, the representative biomedical applications of enzymatically crosslinked-injectable hydrogels are presented, including those for wound healing, preventing post-operative adhesion (POA), and hemostasis. Furthermore, hydrogel composites containing filler materials, such as therapeutic cells, proteins, and drugs, are analyzed. In conclusion, we examine the scientific challenges and directions for future developments in the field of enzymatically crosslinked-injectable hydrogels, focusing on material selection, intrinsic properties, and filler integration.

## 1. Introduction

Hydrogels are three-dimensional (3D) crosslinked networks of hydrophilic polymers with capacity for high water uptake [1]. Owing to their hydrophilic characteristics, softness, excellent biocompatibility, and structural similarities to tissues, hydrogels have been spotlighted as matrices for use in various biomedical applications [2,3], e.g., medical device interfaces [4,5,6], 3D cell culture platforms [7], bio-ink materials for use in 3D printing [8,9,10], conductive contact electrodes [11,12,13,14], and filler delivery reservoirs [15,16]. Conventional hydrogels are generally synthesized in a laboratory and subsequently implanted into the patient via invasive surgical procedures [17], posing a significant burden on patients. Furthermore, a comprehensive understanding of the geometry of the target site is essential before the hydrogel implantation, complicating the implantation and operation processes [18].

**Figure 1:**
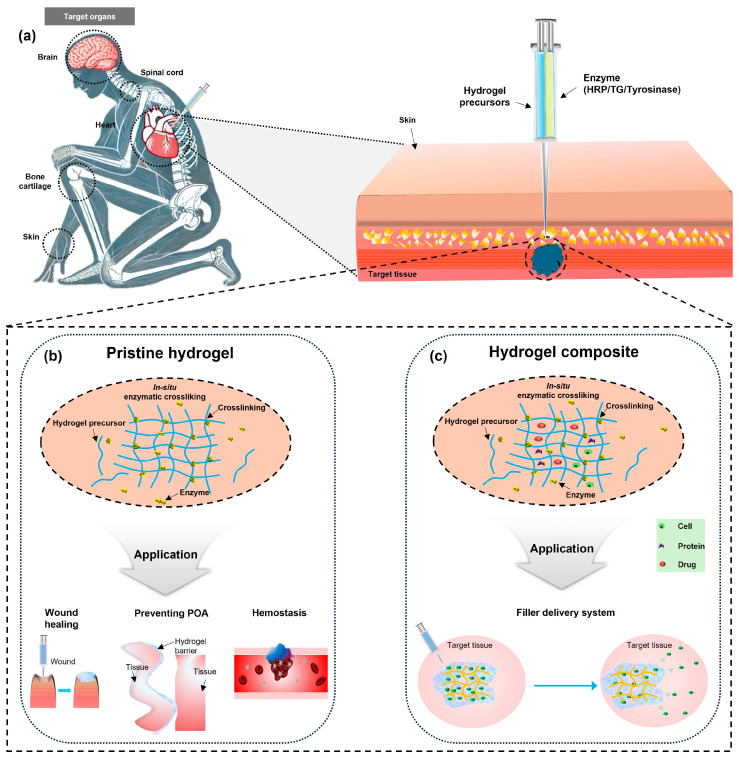
Schematic diagram of an enzymatically crosslinked injectable hydrogel. (**a**) Potential applicable organs for the use of injectable hydrogels (**left**) and their minimally invasive administration protocol whereby hydrogel precursors and enzymes are injected a via a dual syringe (**right**). (**b**) Enzymatic gelation in situ and the applications of pristine hydrogels. (**c**) Enzymatic gelation of hydrogel composites in situ with various therapeutic fillers including cells, proteins, and drugs.

Injectable hydrogels possess unique properties that enable them to transform from a precursor solution state to a gel state in situ [19]. Due to their distinctive minimally invasive administration, injectable hydrogels offer several inherent advantages, e.g., the significant alleviation of patient discomfort (Figure 1a) [17]. In addition, injectable hydrogels can form seamless integrations with curvilinear tissue surfaces, which is critical in enhancing the performances of the hydrogels by facilitating tissue–device interactions [18,20].

Hydrogels can be classified into two types based on their crosslinking mechanisms: hydrogels with covalent crosslinking, i.e., chemical hydrogels, and hydrogels with noncovalent interactions, i.e., physical hydrogels [21]. Although physical hydrogels can be formed under mild conditions without the aid of catalysts, their low mechanical properties limit their wider applicability. On the other hand, chemical hydrogels exhibit high stability and tunable mechanical strengths. However, conventional chemical crosslinking methods often hamper their bioavailability owing to the utilization of harmful substances (e.g., chemical catalyst [22]) or extreme reaction conditions (e.g., high temperature [23] and ultraviolet light [24]). Therefore, biocompatible chemical crosslinking methods, e.g., via click chemistry [25], have been spotlighted to avoid such issues.

Enzymatically crosslinked hydrogels are also an example that could provide the advantages of chemical hydrogels, while minimizing the disadvantages by using enzymes as crosslinking mediators [2]. The gelation time, mechanical properties, and physical properties of the hydrogels can be easily controlled by modulating the concentrations of polymer chains and enzymes, and conjugation degree of functional moieties [26,27]. As enzymes naturally exist in the body, the biocompatibility of enzymatically crosslinked-hydrogels is superior to that of chemical hydrogels crosslinked via other methods [2], such as photo-crosslinking, thermal-crosslinking, or chemical catalyst-aided crosslinking. Enzymatic crosslinking does not require any toxic reagents or harsh reaction conditions, such as ultraviolet light irradiation that may damage normal tissues [24]. Enzyme-mediated crosslinking can occur at physiological pH and temperature, enhancing their practicality for biomedical applications [26]. Moreover, widely used natural polymers (e.g., gelatin [28], hyaluronic acid [29]) and synthetic polymers (e.g., polyethylene glycol (PEG) [30]) applications can be employed for enzyme-mediated crosslinking, indicating the versatility of enzymatically crosslinked injectable hydrogels.

In this review, we address various types of enzymatic crosslinked injectable hydrogels depending on the enzyme type and describe material properties and applications of such representative ones. The enzymatically crosslinked injectable hydrogels simultaneously combine both advantages of injectable and enzymatically crosslinked hydrogels. Typical injectable enzymatic crosslinking mechanisms, e.g., horseradish peroxidase (HRP)-, transglutaminase (TG)-, and tyrosinase-mediated crosslinking are presented, followed by an analysis of several factors that control the intrinsic properties of the hydrogel. We then discuss the biomedical applications of enzymatically crosslinked injectable hydrogels, as pristine hydrogels and hydrogel composites integrated with functional filler materials. Pristine hydrogels can be applied for wound healing, prevention of post-operative adhesion (POA), and hemostasis (Figure 1b). Hydrogel composites can be used to deliver therapeutic agents, including cells, proteins, and drugs (Figure 1c). Finally, this review addresses the technical pathways and remaining challenges of enzymatically crosslinked-injectable hydrogels in terms of material selection, intrinsic properties, and filler integration.

## 2. Enzymatic Crosslinking Mechanisms

Enzymatic crosslinking has garnered significant attention due to its higher biocompatibility and tunable properties associated with its mild reaction conditions and the controllability of the gelation reaction, compared to many classical chemical crosslinkers [2,26]. In this section, we introduce the enzymes that are generally used as crosslinkers in injectable hydrogels. Many types of enzymes have been utilized, such as sortases, oxidases, transferases, and phosphatases or kinase derivatives [31,32]; however, we focus on HRP, TG, and tyrosinase, which are mainly used in enzymatically crosslinked injectable hydrogels.

**Figure 2:**
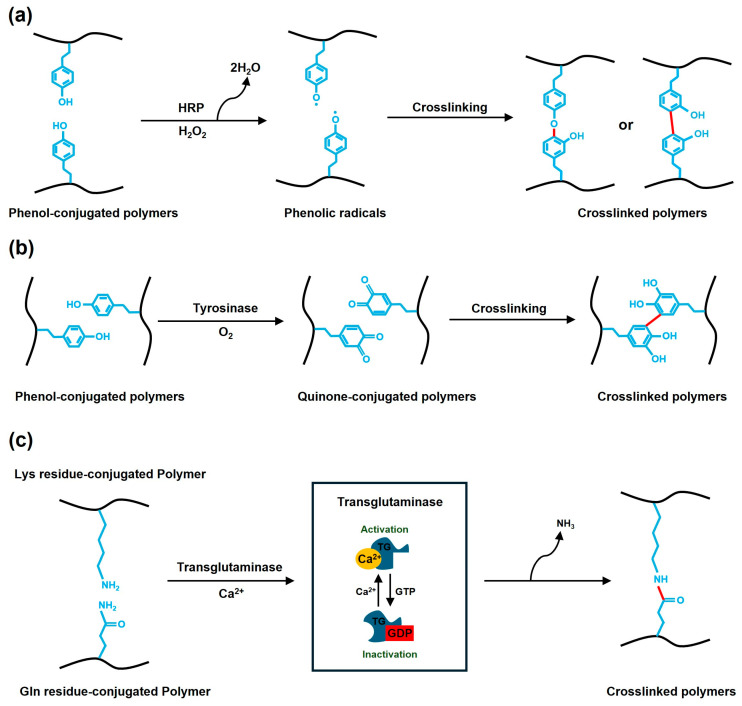
Schematics of the enzymatic crosslinking mechanisms. Mechanisms of crosslinking between (**a**,**b**) phenol-conjugated polymers using (**a**) HRP/H_2_O_2_ or (**b**) tyrosinase and (**c**) primary amine- and γ-carboxylamide-linked polymers using tissue TG can be utilized to fabricate hydrogels.

HRP, naturally found in the root of horseradish, is the most commonly used enzyme in hydrogel synthesis [33]. HRP reacts with H_2_O_2_ to convert phenols to phenolic radicals, producing H_2_O as a byproduct, and the phenolic radical precursors are crosslinked with each other only at the target site (Figure 2a), enhancing injectability [34]. Moreover, the use of HRP in combination with H_2_O_2_ enables more precise control of the gel properties, as two crosslinking agents are involved. To increase the crosslinking density, several hydrogel matrix polymers (e.g., gelatin, chitosan, hyaluronic acid, PEG, and dextran) can be conjugated with phenol derivatives such as tyramine or hydroxyphenylpropionic acid, to facilitate the optimization of the mechanical properties of the hydrogel [33].

However, the presence of H_2_O_2_, which is classified as a reactive oxygen species (ROS), may compromise biocompatibility, leading to tissue necrosis, inflammation, and other harmful effects [35]. To address the problems associated with direct H_2_O_2_ injection, novel approaches that facilitate in situ H_2_O_2_ generation via dual-enzyme systems, such as HRP/glucose oxidase (GOx) or HRP/galactose oxidase (GalOx), have been proposed. GOx and GalOx oxidize monosaccharides within the target tissue, generating H_2_O_2_ in situ as byproducts [28,36,37]. Nevertheless, this approach still faces limitations, such as cytotoxicity, due to overproduction of H_2_O_2_ [38], highlighting the need for an H_2_O_2_-free crosslinking mechanism.

Tyrosinase is a metalloenzyme that contains copper ions. It can be extracted from natural sources such as mushrooms or recombinant *Streptomyces avermitilis* [39]. Tyrosinase is a polyphenol oxidase that oxidizes substrates to phenolic moieties using copper ions and oxygen [39,40]. During crosslinking, phenolic substrates oxidized by tyrosinase are converted to quinones via phenoxides (Figure 2b) [39]. Quinones are highly reactive compounds that can combine with other phenols or amines to form crosslinks between polymer chains [39,40,41]. In addition, quinones display high reactivity toward amine, thiol, and alcohol groups on tissue surfaces, which can impart enhanced adhesion to hydrogels [42]. This mechanism mediates the reactions between polymers with phenolic moieties (e.g., gelatin, collagen, fibronectin, and casein) similarly to HRP, but it is more biocompatible because it consumes oxygen instead of ROS during oxidation [39,43]. Polymers (e.g., hyaluronic acid, chitosan, and alginate) can be modified with phenolic motifs such as 3,4-dihydroxyphenylalanine or tyramine [44], to adjust the crosslinking density.

TGs catalyze the covalent bonding between the γ-carboxylamide groups of glutamine residues and ε-amino groups of lysine residues (Figure 2c) [45]. These bonds are highly resistant to proteolytic degradation, resulting in stable polymeric networks [32]. Similar to the HRP crosslinking system, polymers (e.g., hyaluronic acid and PEG) can be modified with primary amines and glutamine residues to increase the crosslinking density [46,47,48]. Additionally, the use of natural polypeptides, such as gelatin, fibrin, and soy proteins, which contain amino acids such as lysine and glutamine, can obviate the need of polymer modifications for crosslinking [48]. TG mainly originates from mammals and microorganisms, tissue TG (tTG) and microbial TG (mTG) are commonly used enzymatic crosslinkers in injectable hydrogel synthesis [39,48].

tTG, which is derived from animal tissues, exhibits optimal activity under physiological conditions, rendering it highly biocompatible [32]. tTG requires calcium for enzyme activation, which involves the exchange between guanosine triphosphate (GPT) and guanosine diphosphate (GDP). This need for precise calcium regulation in the reaction environment can limit its applications [45]. In contrast, mTG is mainly derived from microorganisms and its mass production is cost-effective [48]. Compared to tTG with complex multidomain structures, mTG is a simple single polypeptide structure and is calcium-independent because it does not contain calcium-binding domains [49]. Due to these structural differences, mTG exhibits optimal activity in a broad pH range (5–8) and at temperatures of 40–50 °C, indicating their potential broader application scope than that of tTG [48,49,50].

## 3. Control of Hydrogel Physicochemical Properties

In this section, we introduce methods for preparing enzymatically crosslinked injectable hydrogels with the desired physicochemical properties. Controlling the properties of the hydrogel after crosslinking reaction that occurs during injection is essential to ensure high efficacy and safety in biomedical applications [16,18,51]. It also allows for optimized enzyme activity, biocompatibility, and mechanical properties, controlled filler release, predictable degradation, and favorable cellular interactions [27,33,51,52]. The physicochemical properties of enzymatically crosslinked injectable hydrogels, including their mechanical strength, gelation and degradation rates, swelling ratio, and pore size, can be finely tuned by adjusting the concentrations of the hydrogel precursors, enzymes, and oxidizing agents (Table 1) [31,32].

### 3.1. Modulus

The modulus value of a hydrogel plays a crucial role in various biomedical applications as it affects the material’s cohesion, physical stability, and interactions with human tissues [53,54]. A mechanical mismatch between the hydrogel and tissue may induce an inflammation reaction and degradation of performance [55,56,57,58]. Furthermore, the modulus significantly affects various cellular behaviors via dynamic mechanical signals that are transferred to the nuclei by cell surface receptors such as integrins, leading to corresponding gene expression [27,33,59,60].

As the crosslinking density increases, the modulus also increases. The crosslinking density is proportional to the concentration of hydrogel precursor [61,62,63,64,65], crosslinking enzyme [28,61,62], and oxidizing agent [36,62,65,66,67]; thus, these concentrations are also proportional to the modulus. In the case of HRP-mediated crosslinking, exceeding the optimal range of H_2_O_2_ concentration results in low crosslinking density owing to the inhibition of HRP activity by excess H_2_O_2_ [62]; thus, experimentally identifying the optimal concentration of H_2_O_2_ is critical.

### 3.2. Gelation Rate

The gelation rate of an injectable hydrogel is critical. If the gelation rate is excessively high, the gel formation can occur within the injector, thus affecting the mechanical properties. If the gelation rate is too slow, the fillers or hydrogel precursors may be lost in vivo [18,37]. The gelation rate is also related with the filling of curvilinear tissue defects, leading to conformal interfacing [33,51].

The gelation rate is proportional to the concentrations of the hydrogel precursor, crosslinking enzyme, and oxidizing agent, based on the initial stoichiometric ratio and chemical kinetics [68,69]. In the case of HRP-mediated crosslinking, exceeding the optimal range of H_2_O_2_ concentrations will result in a prolonged gelation time owing to the inhibition of the HRP-activity by excess H_2_O_2_ [62].

### 3.3. Degradation Rate

The degradation rate of a hydrogel ensures biosafety and convenience for patients by preventing long-term inflammatory problems and eliminating the need for additional removal procedures [70,71,72,73]. In filler delivery, in particular, the release rate of fillers within the hydrogel is highly related to its degradation behavior [33].

Generally, a higher crosslinking density in a hydrogel generates a denser mesh structure, which results in a slower degradation rate [68]. Therefore, the degradation rate is inversely proportional to the concentrations of the hydrogel precursor [37,61,62,63,74], crosslinking enzyme [61], and oxidizing agent [67]. Additionally, the degradation of natural polymer-based hydrogels can be facilitated using degradation enzymes, e.g., hyaluronidase. Thus, tailoring the concentration of the degradation enzyme can regulate the degradation rate and affect the filler release behavior [75].

### 3.4. Swelling Ratio

The swelling ratio reflects the capacity to absorb and retain water, and the water uptake capacity is linked to the delivery of fillers from hydrogels [30]. Furthermore, volumetric changes associated with swelling can affect hydrogel stability [37]. Thus, the optimal swelling ratios of hydrogels should be determined for biomedical applications.

The factors affecting the manipulation of the swelling ratio vary depending on the specific case. Several studies suggest that increasing the polymer concentration results in a higher swelling ratio [37], whereas others advocate for a reduction in the concentration [63,74]. The tendency of the swelling ratio is influenced by characteristics of the hydrogel polymer such as hydrophobicity [76], because the swelling ratio is highly related to the water absorption capacity.

### 3.5. Pore Size

The pore size of a hydrogel is closely related to its capacity to absorb water [30], in addition to its capacities to retain and release fillers and control cell behavior [77,78,79]. Hydrogels with small pores hamper cell migration, which is crucial in tissue regeneration; however, those with large pores lead to burst release of small fillers [1,80,81]. Therefore, tailoring the pore size to suit the desired application is crucial

The pore size of a hydrogel is inversely proportional to the crosslinking density [45,82]; thus, the pore size is inversely proportional to the concentrations of hydrogel precursor [39,83,84,85], enzyme [86], and oxidizing agents [67]. The correlations between the physicochemical properties and various parameters are summarized in Table 1.

However, the sophisticated modulation of the physicochemical properties of pristine hydrogels by adjusting only these parameters is challenging. Therefore, recent research efforts focused on controlling the crosslinking density by incorporating nanocomposites such as metal nanoparticles (NPs), which induce additional interactions (e.g., ionic and coordination bonds), to adjust various physicochemical properties [87,88,89]. Such interactions can enable the successful design of precise enzymatically crosslinked injectable hydrogels for use in the desired applications.

**Table 1:** Characteristics of the enzymatically crosslinked injectable hydrogels and their modulation by varying the concentrations of the reactants.

	Hydrogel Properties	Modulus	Gelation Rate	Degradation Rate	Swelling Ratio	Pore Size
Reactant						
Enzyme		Proportional [28,61,62]	Proportional [28,62,63,66,67]	Inverse [61]	Inverse [90]	Inverse [86]
Polymer		Proportional [61,62,63,64,65]	Proportional [67,74]	Inverse [37,61,62,63,74]	Proportional [37] Inverse [63,74]	Inverse [39,83,84,85]
Oxidizing agent		Proportional [36,62,65,66,67]	Proportional [36]	Inverse [67]	Inverse [67]	Inverse [67]

## 4. Applications of Enzymatically Crosslinked Injectable Hydrogels

The hydrophilic properties and 3D structure of a hydrogel are closely related to the structure of the natural extracellular matrix, rendering the pristine hydrogel suitable for use in various biomedical applications (e.g., biological scaffolds and wound dressings) [91]. Such relevance, with additional imparted features, has resulted in commercialization [92]. Considering their distinctive advantages of biocompatibility and injectability, enzymatically crosslinked injectable hydrogels have also been widely studied owing to their capacities to leverage the inherent properties of pristine hydrogels. This section examines three prevalent applications of enzymatically crosslinked injectable hydrogels: wound dressing, POA prevention, and hemostasis.

### 4.1. Wound Healing

**Figure 3:**
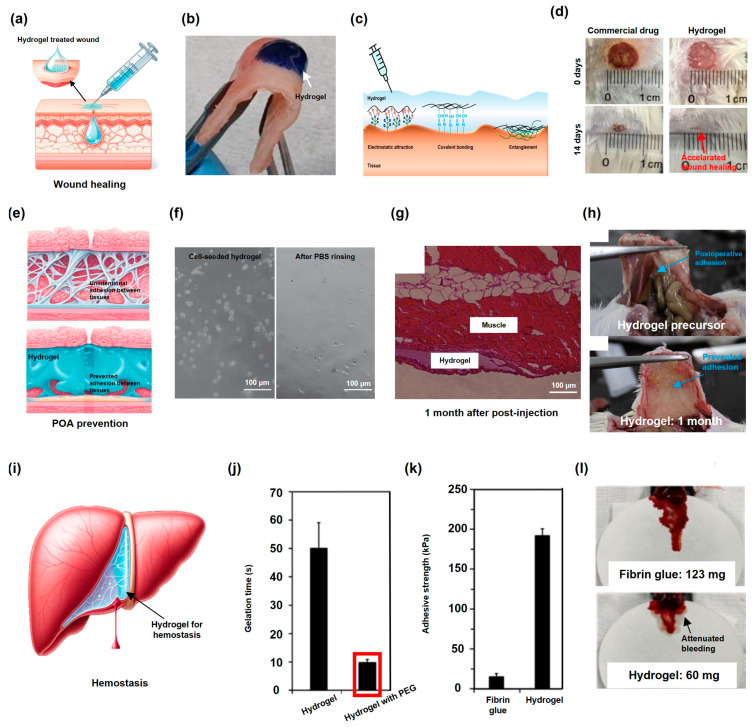
(**a**) Hydrogel application as a wound dressing. (**b**) Conformal integration of a hydrogel with porcine skin, despite deformation (reused with permission [29]). (**c**) Three different strategies that can enhance the levels of tissue adhesiveness of hydrogels. (**d**) Superior wound healing efficacy of an enzymatically crosslinked injectable hydrogel (**right**) compared to that of a commercial drug (**left**) (reused with permission [97]). (**e**) Mechanism of POA prevention of a hydrogel as a physical barrier. Unintentional adhesion between tissues (**top**). Hydrogel injected between tissues prevents tissue adhesion (**bottom**). (**f**) Cells within hydrogels rinsed off due to low levels of adhesion before (**left**) and after rinsing (**right**). (**g**) The hydrogel lasts for a month. (**h**) Prevented POA using an enzymatically crosslinked injectable hydrogel after surgery. Treatment using the hydrogel precursor (**top**) and hydrogel (**bottom**) (figures (**f**–**h**) are reused with permission [61]). (**i**) Hemostasis of the liver achieved using a hydrogel. (**j**) Rapid gelation of the hydrogel compared to that of the hydrogel without PEG. (**k**) Hydrogel exhibits significantly high adhesion strength compared to that of commercial fibrin glue. (**l**) Superior hemostatic capacity of an enzymatically crosslinked injectable hydrogel compared to that of commercial fibrin glue. The levels of blood loss of fibrin glue- (**top**) and hydrogel-treated mice (**bottom**) (figures (**j**–**l**) are reused with permission [30]).

Hydrogels exhibit desirable intrinsic properties, including good moisture retention and the prevention of external bacterial infections by providing physical barriers, rendering them attractive options for use in wound dressings (Figure 3a). The desired functions imparted to hydrogels can boast their efficiencies by guiding skin regeneration and accelerating wound healing [93]. Injectable hydrogels that form gels in situ and completely fill the curvilinear defect areas can promote wound healing with superior efficacies [94]. Furthermore, adhesion on the wound site of hydrogels accelerates wound healing. Strong adhesion at the wound site can prevent accidental hydrogel removal, even with the frequent motions of wounds in movable parts, thereby promoting wound healing [95].

Various strategies have been employed to enhance the levels of adhesion of hydrogels for use in wound dressing. Chitosan can strongly interact with negatively charged human tissues because of its positive charge under physiological conditions, which leads to strong tissue adhesion. Moreover, oxidized chitosan exhibits aldehyde groups that can enhance the adhesion strength via chemical interactions with tissue amines. Oxidized chitosan-based hydrogels show superior adhesion strengths on porcine skin compared to that of fibrin glue, leading to effective wound sealing and accelerated healing efficiencies in rabbit models [96]. Oxidized hyaluronic acid also contains aldehyde moieties that can enhance the adhesion strength of the hydrogel (Figure 3b). Moreover, the use of long viscous polymer chains can improve the adhesiveness via entanglement with the tissue surface (Figure 3c). The combination of oxidized hyaluronic acid and long viscous polymer results in a double-network hydrogel with a high adhesion strength and an accelerated wound healing efficacy [29].

HRP-mediated crosslinked hydrogels with the oxidizing agent H_2_O_2_ display intrinsic antibacterial properties that can promote wound healing and prevent inflammation. Furthermore, collagen, which is a crucial component in wound healing, provides a scaffold for cell growth, thereby promoting tissue regeneration. Consequently, the HRP-mediated crosslinked collagen-based hydrogel, owing to its antibacterial properties and tissue regeneration capacity, exhibits superior wound healing efficacy compared to that of conventional commercial treatment in mouse models (Figure 3d) [97].

### 4.2. POA Prevention

POAs, which are undesirable tissue adhesions after surgery, pose significant pathological risks [98]. These adhesions can disrupt physiological and mechanical organ functions [99]. Hydrogels have emerged as a promising solution for use in POA treatment because of their capacities to act as physical barriers, preventing adhesion, while minimizing adverse effects on healing after surgery (Figure 3e) [100]. Injectable hydrogels can seamlessly cover curvilinear wounds via minimally invasive administration, displaying significant potential for use in anti-tissue adhesion techniques [101]. These in situ-forming hydrogels effectively prevent POAs compared to conventional treatments, thereby eliminating the need for secondary surgeries to remove POAs [102].

To inhibit fibrotic reactions to the implanted hydrogels, hyaluronic acid-based hydrogels are employed because of their low levels of cell adhesiveness, thereby enhancing their capacities to prevent POAs (Figure 3f) [61]. This characteristic may be attributed to the absence of cell-binding motifs within hyaluronic acid [74]. Such a hydrogel remains at the target site and serves as a physical barrier for a prolonged period (Figure 3g), surpassing conventional treatments with short retention times [101] and exhibiting effective POA prevention in mouse models (Figure 3h) [61].

Soft hydrogels often struggle to adhere to damaged sites, reducing their degrees of effectiveness in preventing POAs, and carboxymethyl cellulose (CMC)-based hydrogels have been used to overcome this limitation. CMC is an ideal polymer for use in enhancing the mechanical properties and preparing a hydrogel with the desired viscoelastic behavior that can fix its position on the target site to effectively prevent POA [103]. In addition, pullulan, which is another viscous polymer, displays superior adhesion properties. Pullulan-based hydrogels exhibit effective POA prevention with high adhesion strengths [104]. Incorporating pullulan into hydrogels can enhance their levels of adhesiveness, enabling them to remain at their target sites despite gravity and organ motion, as demonstrated in rat models [103].

### 4.3. Hemostasis

The uncontrollable bleeding that occurs after trauma or surgery is a leading cause of global mortality [105]. Moreover, the process of regeneration following injury is closely linked to the process of hemostasis [106], further indicating the importance of controlling bleeding. Injectable hydrogels present outstanding features for use in hemostasis, including their capacities to reach deep bleeding wounds with reduced pain, thereby facilitating hemostasis and promoting the regeneration of injured tissue (Figure 3i) [107].

A positively charged polymer-based hydrogel exhibits promise as an effective hemostatic material owing to its strong degrees of adhesion to negatively charged tissues, leading to adherence to damaged sites and the prevention of bleeding. Furthermore, the rapid gelation of the hydrogel within seconds further enhances its hemostatic efficiency. ε-Polylysine displays superior adhesion properties due to its cationic primary amine groups, indicating that it is a suitable material for use in designing hemostatic hydrogels. A ε-polylysine-based hydrogel exhibits superior adhesion than commercial fibrin glue with a rapid gelation rate, followed by the effective management of a hepatic hemorrhage [67].

However, an excess of positive charges on the polymer can hamper its biocompatibility owing to charge imbalance. To address this problem, PEG grafting onto polymers can be used to reduce their positive charges and decrease their cytotoxicity [67]. Moreover, PEG can be used to enhance the mechanical properties and adhesive strengths of hydrogels, thereby increasing their hemostatic capacities. The rapid gelation of a hydrogel was realized by varying the molecular weights and structures of the PEG chains (Figure 3j), and the mechanical properties and adhesive strength were further optimized by enhancing the cohesive strength (Figure 3k). Thus, the strategic employment of PEG not only improves the structural integrities of hydrogels, but also significantly enhances their functionalities as hemostatic materials, surpassing the effectiveness of fibrin glue (Figure 3l) [30,108].

Using TG as a crosslinking agent can significantly enhance the hemostatic properties of hydrogels, because TG is a natural blood coagulation factor [109]. TG crosslinks coagulation-related proteins, such as fibrin, promote the formation of blood clots [110,111,112,113]. Additionally, in situ-forming hydrogels that utilize highly expressed TG at bleeding sites have demonstrated effective hemostatic ability, further indicating the promise of TG as a hemostatic agent [114].

## 5. Applications of Enzymatically Crosslinked-Injectable Hydrogel Composites

The filler integration with hydrogels can provide additional functionalities, such as stimuli-responsive behavior, conductivity, and non-invasive imaging capacity, which can broaden their application scope in biomedical fields [88,115,116,117]. Among these, hydrogels exhibit significant promise as delivery reservoirs for therapeutic fillers [88,118]. The local administration of fillers using hydrogels can overcome the limitations of systemic administration, such as a low targeting efficiency and the short half-lives of the fillers [119]. Moreover, the use of hydrogels can reduce the frequency of infusions [120,121] and prevent the undesirable leakage of fillers into healthy tissues [122].

Cells, proteins, and drugs can be incorporated into hydrogels as therapeutic agents. As cellular vehicles, hydrogels permit the free exchange of metabolites while protecting encapsulated cells from immune attacks, cytotoxic molecules, and mechanical stress, thereby enhancing cell survival [123,124]. Hydrogels can also protect protein structures, maintaining their functionality [125,126], and enhance the bioavailability and solubility of drugs with effective loading capacity [120,127]. Furthermore, as sustained release systems, hydrogels can ensure the appropriate therapeutic doses and frequencies of fillers, which are crucial in the fields of cell, protein, and drug therapies [125,126,128,129,130,131,132].

Injectable hydrogels, as drug delivery vehicles, enable the delivery of fillers to hard-to-reach tissue sites [122] via minimally invasive procedures, thereby significantly reducing patient discomfort [125,126]. Enzymatically crosslinked injectable hydrogels, with excellent biocompatibility, have been extensively studied as filler delivery reservoirs. These in situ-forming hydrogels do not require harsh exogenous energy supplies or toxic materials for gelation, thereby enhancing their potential as delivery reservoirs for therapeutic agents.

### 5.1. Cell-Hydrogel Composites

**Figure 4:**
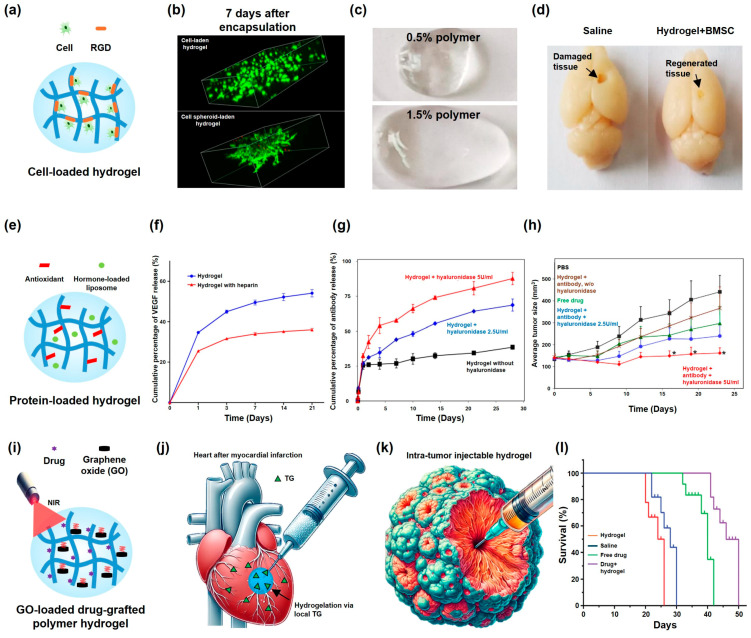
(**a**) Cell-loaded hydrogel composite with RGD moieties, which are cell binding motifs. (**b**) Live/dead assay of cell (**top**) and cell spheroid-laden (**bottom**) hydrogels (reused with permission [133]). (**c**) Images of hydrogels with different swelling ratios after soaking in phosphate-buffered saline for 24 h. Swollen hydrogels with low (**top**) and high (**bottom**) concentrations of the polymer. (**d**) Accelerated healing of a TBI using an enzymatically crosslinked injectable hydrogel (**right**) compared to that observed in the control group (**left**) (figures (**c**,**d**) are reused with permission [37]). (**e**) Functional protein recombinant hormone-loaded liposome loaded into a polymer hydrogel. (**f**) Cumulative levels of VEGF release from a hydrogel (blue) and hydrogel with heparin (red) (reused with permission [62]). (**g**) Cumulative release profiles of antibody-loaded hydrogels based on the hyaluronidase concentration. (**h**) Time lapse changes in tumor volume observed using various antibody-loaded enzymatically crosslinked injectable hydrogels (figures (**g**,**h**) are used with permission [75]). (**i**) Drug-grafted polymer hydrogel with graphene oxide (GO). Under near-infrared (NIR) light, the GO within the hydrogel can generate heat. (**j**) Gelation of the hydrogel due to locally overexpressed TG in myocardial infarction (MI). (**k**) Intra-tumoral injection of the enzymatically crosslinked injectable hydrogel. (**l**) Survival rates observed using the drug–hydrogel composites in mouse tumor models (reused with permission [148]).

Cell-laden hydrogels can enhance wound healing efficiency. Gelatin-based hydrogels are widely used in cell encapsulation owing to their intrinsic properties, such as low antigenicity and the presence of cell adhesion motifs (Figure 4a). These features contribute to the superior wound healing efficacy of cell-laden gelatin-based hydrogels compared to those of conventional cell-based treatments [28]. Furthermore, 3D cell spheroids were effectively incorporated into the hydrogel matrix (Figure 4b), leading to superior healing effects in burn wounds in a murine model. Cell spheroids more closely mimic the natural physiological conditions of tissues than conventional 2D cultured cells, suggesting the higher regenerative potential of the 3D cell spheroid–hydrogel composite [133].

Cell-laden hydrogels have been studied for the regeneration of the central nervous system. The HRP/GalOx dual enzymatically crosslinked hydrogel can enhance the growth and neuronal differentiation of mesenchymal stem cells, thereby optimizing the efficiency of cell-based therapy in spinal cord injury repair in mouse models [36]. In another study, the backbone polymer of a hydrogel was modified with an RGD sequence, which enhanced the cell adhesion and improved cell survival and growth. The RGD-functionalized hydrogel, when incorporated with desired cells, displayed efficacy in promoting spinal cord repair in a rat model [55].

HRP/GOx-mediated crosslinked hydrogels containing cells also exhibited superior effects in healing traumatic brain injuries (TBIs). The concentration of GOx is optimized to ensure a high cytocompatibility, maintaining the concentration of generated H_2_O_2_ to avoid its adverse effects on cell behavior [77]. However, the swelling behavior of hydrogel may damage the surrounding tissues, which are critical in the nervous system. To minimize this effect, a hydrogel with an anti-swelling behavior was explored by varying the polymer concentrations (Figure 4c). The resultant optimized hydrogel, which is integrated with cells, facilitates the survival and proliferation of endogenous neural cells, resulting in the effective healing of TBI (Figure 4d) [37].

Inadequate vascularization can lead to tissue ischemia [134], which is a major hurdle in tissue implantation. A seamless interface between the hydrogel and recipient tissue can effectively induce neovascularization, rendering injectable hydrogels promising materials for use in promoting the formation of new blood vessels [135]. Gelatin-based hydrogels, based on their advantages, such as their cell-binding motifs, significantly enhance the growth of encapsulated cells, positioning them as promising candidates for use in vascularization. The improved retention of the encapsulated cells leads to successful neovascularization [136]. Collagen hydrogels with abundant cell-binding motifs, similar to gelatin, have been shown to promote angiogenesis depending on the encapsulated cell types [63]. Such property can be imparted to hydrogels composed of polymers lacking cell-binding motifs, such as hyaluronic acid, by introducing RGD moieties, which enables the co-utilization of human umbilical vein endothelial cells and fibroblasts by enhancing cell attachment. The efficacy of this method induces the formation of functional vasculature in subcutaneous tissue in mouse models, which is superior to those observed using single cell-laden hydrogels [137].

### 5.2. Protein-Hydrogel Composites

Bone morphogenetic protein-2 (BMP-2) loaded into enzymatically crosslinked-injectable hydrogels exhibits an excellent bone regeneration capacity. Hyaluronic acid hydrogels, which enhance the BMP-2 bioactivity by facilitating the recruitment of the BMP-2 receptor subunit [138], were used to promote osteogenesis in mouse models [139]. Moreover, the hydrogel exhibited adhesive properties via electrostatic attraction to the target site, which facilitated effective calvarial bone healing with the aid of a recombinant BMP-2 filler [64]. This in situ forming hydrogel holds promise for use in the minimally invasive treatment of cranial injuries, by simplifying the complex process of bone regeneration [140]. In another approach, integrating recombinant hormone-loaded liposomes, which induce cartilage regeneration to heal osteoarthritis, into an antioxidant-grafted polymer hydrogel (Figure 4e) successfully cured osteoarthritis in mouse defect models [141].

Vascular endothelial growth factor (VEGF)-loaded hydrogels induce neovascularization. Gelatin, which inherently contains numerous primary amines and carboxylic acid groups, has been used in designing hydrogels. These functional groups can be easily conjugated to molecules containing crosslinking moieties, such as tyramine. Similarly, heparin can be grafted into gelatin, which provides a binding site for the growth factor, thereby protecting it from degradation. This leads to the enhanced bioactivity of the loaded VEGF, subsequently prolonging the sustained release of the filler (Figure 4f). These protein–hydrogel composite successfully induced angiogenesis in subcutaneous mouse models [62].

Hydrogels loaded with anticancer proteins have been explored as potential cancer therapeutics. By manipulating their degrees of crosslinking, such composites enable the sustained release of fillers, which is critical in treating chronic cancer [66]. Antibody-loaded hydrogels display excellent tumor-suppressive effects in breast cancer models. Anionic hydrogel polymers were used to prevent the initial burst release of cationic antibodies via electrostatic attraction (Figure 4g). With the aid of hydrogel degradation enzymes, the sustained release of the fillers is observed for over a month, promoting the desired cancer suppression compared to that observed via bare antibody administration (Figure 4h) [75].

### 5.3. Drug–Hydrogel Composites

Gelatin-based hydrogels exhibit intrinsic ROS-scavenging capacities owing to the electron-donating groups on their backbones. Incorporating antioxidants further enhances the ROS-scavenging capability of the hydrogel, thereby improving its effectiveness as a wound dressing. Consequently, skin regeneration promoted by the drug-loaded gelatin-based hydrogel displays a superior wound healing efficacy compared to that of skin regeneration promoted by the pristine hydrogel [142].

Graphene oxide (GO) is a photothermal therapy (PTT) agent used in wound healing. GO-loaded drug-grafted polymer-based hydrogels used in PTT treatment (Figure 4i) exhibit antibacterial and antioxidant properties, thus displaying superior wound healing efficacies [143]. Drug-capped silver NPs (AgNPs) can also be considered as photothermal and antioxidant therapeutic agents for use in wound healing [35].

An enzymatically crosslinked-injectable hydrogel containing drug fillers exhibits therapeutic effects in other target organs, such as cartilage. Drug-loaded hyaluronic acid/gelatin hydrogels display superior cartilage repair capacities in osteoarthritis-induced mouse models [74]. Such a hydrogel enables the sustained release of the drug for up to a month, which is a key in countering musculoskeletal disorders, such as rheumatoid arthritis [144]. Additionally, myocardial infarction (MI) can be repaired using a drug containing NP-hydrogel composite. Polydopamine (PDA) NPs, which exhibit high adsorption capacities for drug molecules, provide the sustained release of drugs, as well as enhance the physical crosslinking of the hydrogel. This hydrogel utilizes locally expressed TGs as chemical crosslinking agents and, thus, the mechanical properties are enhanced in situ (Figure 4j), which is particularly applicable only for injectable hydrogels. Due to these advantages, hydrogels integrated with PDA-drug NPs display sustained drug release and endure the dynamic stress induced by the heart [145].

Injectable anticancer drug hydrogels offer a promising approach for use in local tumor treatment by minimizing the systemic toxicity associated with conventional drug delivery [146]. Injectable hydrogels offer significant advantages in intra-tumoral administration, enabling direct tumor targeting and prolonged drug retention via sustained release (Figure 4k). In mouse models, drug-loaded hydrogels display superior levels of tumor inhibition compared to those of free drugs, with lower systemic toxicities (Figure 4l) [147,148]. However, a sophisticated drug delivery strategy should be developed based on intratumorally injected hydrogels, which differ significantly from in vitro designed hydrogels.

## 6. Conclusions

This review outlined the principles, types, and biomedical applications of enzymatically crosslinked injectable hydrogels. The detailed mechanisms involved in enzymatic crosslinking, e.g., HRP, TG, and tyrosinase mediation, are described. Furthermore, various methods of modulating the hydrogel properties, such as stiffness, gelation/degradation rate, swelling ratio, and pore size, were analyzed. In terms of biomedical applications, we focused on wound healing, POA prevention, and the hemostatic capacities of pristine hydrogels, in addition to the potential of hydrogel composites containing therapeutic fillers (e.g., cells, proteins, and drugs) for use in clinical science.

**Figure 5:**
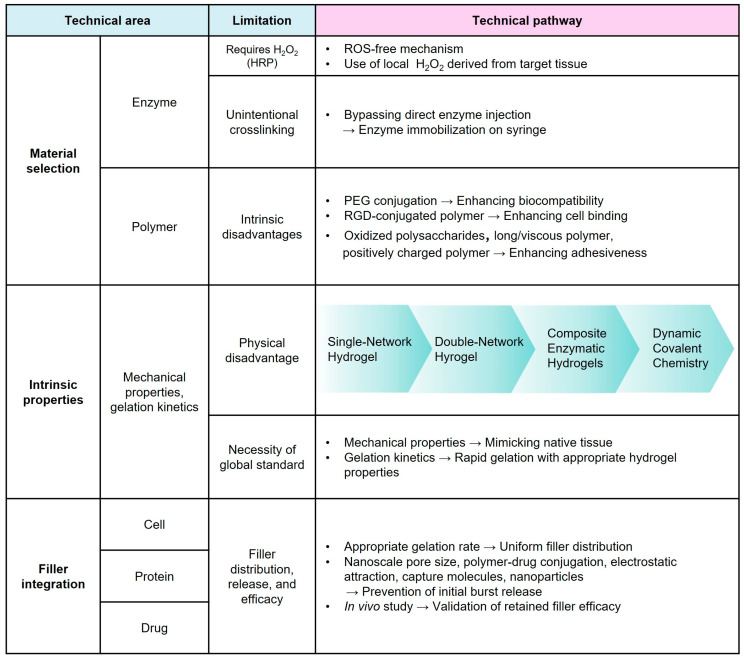
Current scientific challenges and desirable future studies of enzymatically crosslinked injectable hydrogels.

The unique crosslinking mechanisms exhibit significant potential in terms of injectability and biocompatibility; however, several problems must be addressed before commercialization. The distinctive features of the enzymes pose challenges in material selection (Figure 5). Unintentional crosslinking associated with remaining enzymes, such as HRP [149], and the cytotoxicity associated with cofactors, such as H_2_O_2_ [28,35,61], which is involved in the most commonly used enzyme, are the critical factors that compromise the biocompatibility of hydrogels. Therefore, the implementation of the enzyme bead-attached syringe to avoid direct injection of chemicals [149] or the use of local H_2_O_2_ can be suggested as a solution. The polymers should also be modified to overcome limitations. PEG conjugation (i.e., PEGylation) to the backbone polymer of a hydrogel can improve its biocompatibility [67], whereas RGD modification can enhance its cell attachment [55]. Incorporating oxidized polysaccharides [29,96], long/viscous [29,103,104], or positively charged polymers [64,67,96] can improve the adhesiveness of hydrogels.

The mechanical properties of enzymatically crosslinked injectable hydrogels are crucial in designing hydrogels optimized for use in biomedical applications [52,150]. Several strategies can be employed to modulate the mechanical properties, such as double network hydrogels, which contain two polymer networks to enhance their strengths [52,151,152]. Additionally, the incorporation of nanomaterials can further improve the mechanical properties [29,51]. Dynamic covalent chemistry can also be utilized to impart mechanical properties, such as self-healing and anti-fatigue capabilities, to hydrogels [153,154,155]. However, no global standards in terms of mechanical properties have been set, as indicated by the variations in moduli and levels of stretchability across studies [97,143,156,157]. To realize the optimal therapeutic outcomes, future research should focus on selecting hydrogels with appropriate mechanical properties, e.g., mimicking those of native tissues [55,136].

Standards, in terms of not only mechanical properties but also gelation kinetics, which vary across studies, are apparently absent [97,143,156] despite the importance of the gelation rate, which influences the therapeutic efficacy of an injectable hydrogel [58,108]. Rapid gelation is favorable in various clinical cases. This is because it enables the hydrogel to rapidly form a solid state in situ that is fixed at a desired location to induce therapeutic effects and prevent the leakage of fillers from hydrogel composites [32,58].

However, rapid gelation could hinder the uniform distribution of fillers within the hydrogel, thereby affecting the filler release behavior and, thus, optimizing the gelation kinetics is essential [32,58]. The pore size of hydrogels also affects the release of fillers. The mesh sizes of enzymatically crosslinked injectable hydrogels are often larger than the fillers [75], leading to initial burst release [64,68,139]. Scaling down pore size to the nanoscale ensures sustained release, while maintaining the other desirable hydrogel properties. Additional strategies used in preventing burst release exploit drug-grafted polymers [90,142], electrostatic attraction [75], capture molecules [62], and NPs [29,145]. However, such strategies can degrade the therapeutic efficacy of the fillers and, thus, long-term in vivo validation is required. The selection of filler is usually not relevant to the types of polymer or enzyme, but the use of enzyme substrate for filler should be avoided. Nevertheless, chemical bonding between these fillers and the backbone polymer of hydrogels using enzymes can enable the sustained release of therapeutic agents [41]. After addressing the challenges, enzymatically crosslinked injectable hydrogels could be applied in various biomedical fields for clinical translation.

## Data Availability

Not applicable.
